# Supplementary material for: A series of magnon crystals appearing under ultrahigh magnetic fields in a kagomé antiferromagnet
Source: Nat Commun. 2019 Mar 15;10:1229. doi: 10.1038/s41467-019-09063-7 (PMC6420565; doi:10.1038/s41467-019-09063-7)
Supplement: Supplementary file 1 — Supplementary Information [file 41467_2019_9063_MOESM1_ESM.pdf]

Supplementary Information

A series of magnon crystals appearing under ultrahigh magnetic fields in a kagomé antiferromagnet

Okuma et al.,

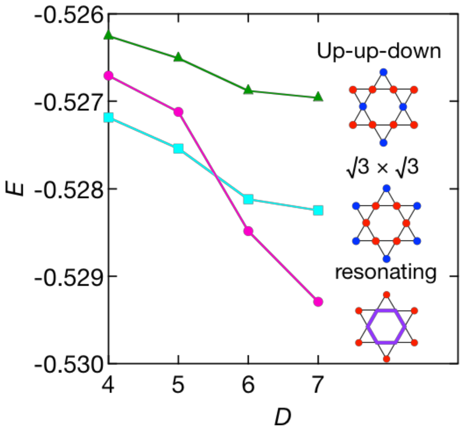

Supplementary Figure 1 Energies at H = 1.1 J calculated by the PEPS method as a function of the bond dimension D for the three spin configurations expected at the 1/3 plateau. In the right side of the figure, the red and blue circles represent up and down spins, respectively, and the purple hexagon represents entangled 6 spins in the singlet state. In the up-up-down and √3 × √3 states, each triangle consists of up, up and down spins with the triangles arranged in **q** = 0 and √3 × √3, respectively, while the resonating state contains a singlet hexagonal magnon. In the left side of the figure, Green triangles, sky blue squares, and violet circles represent the energies of q = 0, √3 × √3, and resonating structures, respectively. Among them, the resonating state finally yields lowest energy as the bond dimension increases.

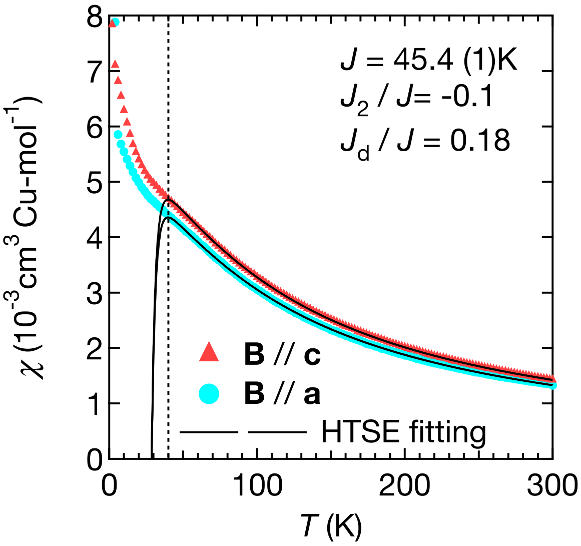

Supplementary Figure 2 Magnetic susceptibility of CdK measured at B = 1 T along the *a* (blue circles) and *c* (red triangles) axes. To estimate the magnitudes of J, J_2 and J_d, both the data above 40 K are simultaneously fitted to the 10th-order-high-temperature-series expansion[45]. The best fit is obtained at (J/K, J_2/J, J_d/J) = (45.4, –0.1, 0.18) with g_a = 2.28 and g_c = 2.37 and a common temperature-independent term χ_0 = –6.65×10^{-5} cm^3 Cu-mol^{-1}. It is noted that these values are approximate as they depend on the temperature region used; the available information from the data is rather limited. Kapellasite is an isostructural compound of CdK that features relative large J_d of 12 K and negligible J_2[1].

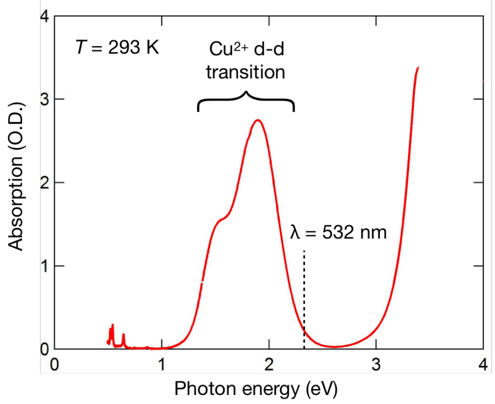

Supplementary Figure 3 Optical absorption spectrum of CdK at room temperature. The red line represents the

observed absorption spectrum. The peak at 1.5–2 eV corresponds to the *d*-*d* transition ($T_2 \rightarrow E$) of Cu^{2+}[2]. Light with λ = 532 nm from a Nd:YAG laser is employed for Faraday rotation measurements, which is located at the bottom of the *d*-*d* transition and thus non-linear effects on the Faraday rotation can be ignored.

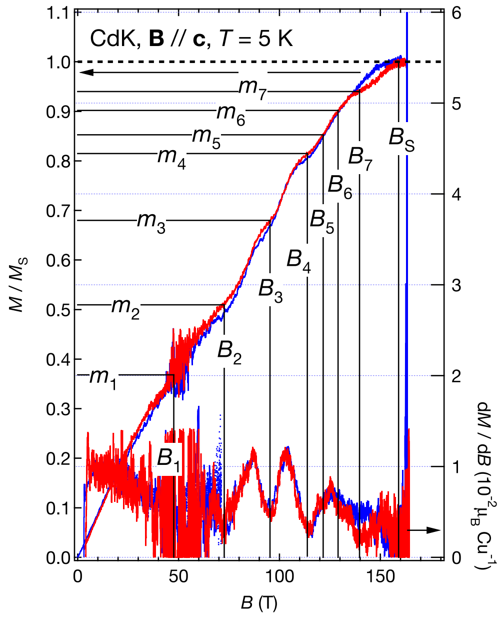

Supplementary Figure 4 Comparison of two successive magnetization measurements with elevating (blue curves) and descending (red curves) magnetic field at 5 K. Anomalies associated with plateaus are observed at almost the same magnetic fields in the two curves. We analyse field-descending data in detail.

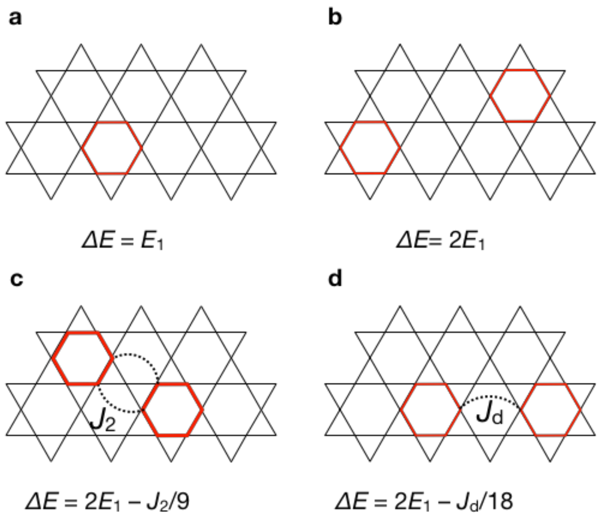

Supplementary Figure 5 Comparison of energies for various magnon configurations. We consider a J–J_2–J_d model in a magnetic field larger than saturation in which the fully polarized state is the ground state as a magnon vacuum with zero energy. **a** the formation of a single hexagonal magnon with S^z = 2 costs $\Delta E = E_1$. **b** $2E_1$ for two independent magnons without mutual interactions. **c** configuration of two nearby magnons expected for Q_{mag} = 9. They are connected by J_2. ΔE is now $2E_1 - J_2/9$. **d** configuration of two nearby magnons for Q_{mag} = 12 with a J_d coupling with $\Delta E = 2E_1 - J_d/18$. A simple argument reveals that antiferromagnetic J_2 and J_d stabilize magnon configurations with Q_{mag} = 9 and 12, respectively, while ferromagnetic interactions lead to destabilization. In CdK, all the configurations with Q_{mag} = 12 are observed, while only on-site 3-magnon configuration with S^z = 0 is observed for Q_{mag} = 9. This fact suggests ferromagnetic J_2 and antiferromagnetic J_d. Magnon crystals with larger unit cells in high fields do not exhibit energy gain or loss by J_2 or J_d and are considered to originate from higher order interactions. We have assigned 19/21 to m_6, not 32/36 and 10/12 to m_5, not 30/36 because adding another magnon to the same hexagon may cost a large on-site repulsion energy in the order of J: it is preferable to increase the density of single magnons as opposed to generating double or triple magnons of less density.

Supplementary References

1. Bernu, B., et al. Exchange energies of kapellasite from high-temperature series analysis of the kagome lattice J_1–J_2–J_d-Heisenberg model. *Phys. Rev. B* **87**, 155107 (2013).
2. Pustogow, A., et al. Nature of optical excitations in the frustrated kagome compound herbertsmithite. *Phys. Rev. B* **96**,

241114 (2017).
